# Cryptococcal 3-Hydroxy Fatty Acids Protect Cells Against Amoebal Phagocytosis

**DOI:** 10.3389/fmicb.2015.01351

**Published:** 2015-12-09

**Authors:** Uju L. Madu, Adepemi O. Ogundeji, Bonang M. Mochochoko, Carolina H. Pohl, Jacobus Albertyn, Chantel W. Swart, J. William Allwood, Andrew D. Southam, Warwick B. Dunn, Robin C. May, Olihile M. Sebolai

**Affiliations:** ^1^Pathogenic Yeast Research Group, Department of Microbial, Biochemical and Food Biotechnology, University of the Free StateBloemfontein, South Africa; ^2^School of Biosciences, University of BirminghamBirmingham, UK; ^3^Institute of Microbiology and Infection and the School of Biosciences, University of BirminghamBirmingham, UK

**Keywords:** 3-Hydroxy fatty acids, Amoeba, *Cryptococcus*, phagocytosis, protection

## Abstract

We previously reported on a 3-hydroxy fatty acid that is secreted via cryptococcal capsular protuberances - possibly to promote pathogenesis and survival. Thus, we investigated the role of this molecule in mediating the fate of *Cryptococcus (C.) neoformans* and the related species *C. gattii* when predated upon by amoebae. We show that this molecule protects cells against the phagocytic effects of amoebae. *C. neoformans* UOFS Y-1378 (which produces 3-hydroxy fatty acids) was less sensitive toward amoebae compared to *C. neoformans* LMPE 046 and *C. gattii* R265 (both do not produce 3-hydroxy fatty acids) and addition of 3-hydroxy fatty acids to *C. neoformans* LMPE 046 and *C. gattii* R265 culture media, causes these strains to become more resistant to amoebal predation. Conversely, addition of aspirin (a 3-hydroxy fatty acid inhibitor) to *C. neoformans* UOFS Y-1378 culture media made cells more susceptible to amoebae. Our data suggest that this molecule is secreted at a high enough concentration to effect intracellular signaling within amoeba, which in turn, promotes fungal survival.

## Introduction

*Cryptococcus* *neoformans* lives primarily in the environment, wherein it is constantly isolated from soil contaminated with bird droppings (Lin and Heitman, 2006). In this ecological niche, cryptococcal cells interact with other organisms, often in a struggle to establish territorial dominance. To illustrate this point, cryptococcal cells have been reported to fall prey to foraging amoebae like *Acanthamoeba castellanii* (Steenburgen and Casadevall, 2003). Additionally, amoebae are said to have evolved efficient strategies to recognize, internalize, and kill internalized microbes, which they use as a source of food (Bottone et al., 1994). And thus the constant struggle between this fungus and amoebae has, as a matter of natural course, selected this fungus to develop a protective structure, i.e., the capsule, in order to evade predation (Kozel and Gotschlich, 1982; Feldmesser et al., 2001). Literature also suggests that cryptococcal cells when under attack from hostile phagocytic cells, including macrophages, express capsule production and enlargement as a defensive mechanism (Steenburgen and Casadevall, 2003; Fuchs and Mylonakis, 2006), because as pointed out by Feldmesser et al. (2001) “from the standpoint of *C*. *neoformans*; there might be little difference between a macrophage and amoeba”… It has been reported that the capsule can enlarge to up to 50 μm in size (Casadevall and Perfect, 1998). Unfortunately this defensive behavior, i.e., capsule production and enlargement, has also translated into this microbe establishing itself as a successful human pathogen, more so in susceptible hosts (Levitz and Boekhout, 2006). During infection, cryptococcal cells can be cleared or can take up residency inside macrophages while avoiding immuno-processing in order to disseminate (Casadevall and Perfect, 1998). Upon being internalized, cryptococcal cells (unlike some pathogenic bacteria) do not prevent fusion of lysosomes to phagosomes (Horwitz, 1983; Voelz and May, 2010), and have adapted to proliferate inside macrophages regardless of the prevailing harsh environment (Levitz et al., 1999). While capsules may be critical in shielding cells (Casadevall and Perfect, 1998), much is still unknown about other metabolites and mechanisms that enable this pathogen to survive when internalized.

We previously reported on the presence and release of a 3-hydroxy fatty acid that is closely associated with capsules of *C. neoformans* UOFS Y-1378 (Sebolai et al., 2007, 2008). 3-Hydroxy fatty acids are regarded as secondary metabolites that have been implicated in the pathogenesis of other microbes (Deva et al., 2000; Ciccoli et al., 2005), but not in *C. neoformans*. Thus, in this study we sought to: (1) estimate the concentration of cryptococcal 3-hydroxy fatty acids being secreted, and (2) investigate the role of these molecules in mediating the fate of cryptococcal cells when acted upon by free-living hostile phagocytic cells.

## Materials and Methods

### Strains, Cultivation and Standardization

The fungal strains, *C. neoformans* UOFS Y-1378 (held at the University of the Free State), *C. neoformans* LMPE 046 (held at the University of the Free State), and *C. gattii* R265 (a gift from R.C. May, University of Birmingham, UK), were maintained on yeast-malt-extract (YM) agar (3 g/l yeast extract, 3 g/l malt extract, 5 g/l peptone, 10 g/l glucose, 16 g/l agar; Merck, South Africa) at 30°C while amoeba, *Acanthamoeba castellanii* LMPE 187 (a gift from A. Idnurm, University of Missouri-Kansas City, USA), was grown on peptone-yeast extract glucose agar, PYG (ATCC medium 30234^TM^) at 30°C. For cryptococcal cells, a loopful of cells was taken from a 48 h old YM agar plate and grown in a 250 ml conical flask containing 100 ml of YNB broth (6.7 g/l; Difco Laboratories, USA) supplemented with 4% (w/v) glucose (Merck) at 30°C for 48 h while agitating at 160 rpm. For amoeba cells, cells were collected from a week old agar plate and cultivated in 50 ml centrifuge tubes (Becton-Dickinson Labware, USA) containing 25 ml of PYG broth at 30°C for 48 h while shaking at 160 rpm. In light of anticipated co-culture experiments, cryptococcal cells were standardized to either 1 × 10^5^ cells/ml, 1 × 10^6^ cells/ml or 1 × 10^7^ cells/ml in 10 ml of either fresh YNB broth or phosphate buffered solution (PBS; Oxoid, South Africa) while amoeba cells were standardized to 1 × 10^5^ cells/ml in 10 ml of fresh PYG broth. All cells were placed on ice before use.

### 3-Hydroxy Fatty Acid Extraction, Analysis and Relative Quantification

3-Hydroxy fatty acids were extracted from 48 h cultures of *C. neoformans* UOFS Y-1378 [cell density after 48 h = 1.7 × 10^7^ cells/ml (±9.0 × 10^5^)] and *C. gattii* R265 [cell density after 48 h = 2.3 × 10^7^ cells/ml (±.6 × 10^5^)] using the modified Folch method. In brief, 2 ml of culture media (containing cells) were transferred to a 15 ml Falcon tube (Becton-Dickinson Labware, USA) following which 2 ml of methanol-chloroform (HPLC-grade) solution (Merck, South Africa; 1:1, v/v) was added. The suspension was vortex mixed and allowed to stand for 20 min. Thereafter, distilled water (2 ml) was added to the above solution and allowed to stand for a further 20 min. The 3-hydroxy fatty acid fraction was collected from the chloroform layer following centrifugation (13000 *g* for 15 min), and was dried under a stream of nitrogen in a fume hood. In a separate experiment, 1 ml of *C. gattii* R265 culture media (48 h) was spiked with 0.5 ml of the 3-hydroxy nonanoic acid 2 mM solution to yield a final concentration of 0.66 mM. Subsequently, 3-hydroxy fatty acids were extracted as detailed above. The analytical 3-hydroxy fatty acid standard viz. 3-OH C9:0, was obtained from Laradon Fine Chemicals (Sweden).

The 3-hydroxy fatty acid extracts (obtained from *C. neoformans* UOFS Y-1378 and *C. gattii* R265) were reconstituted in 50 μl of water, vortex mixed and centrifuged for 15 min at 10000 *g*. The supernatants were transferred to analytical vials with 200 μl fixed inserts and capped (Thermo-Fisher Ltd., UK). The samples were stored in the autosampler at 5°C and analyzed within 72 h of reconstitution in negative electrospray ionisation (ESI) mode. Ultra High Performance Liquid Chromatography-Mass Spectrometry (UHPLC-MS) was performed according to the method reported in Gehmlich et al. (2015), applying a 5 μl sample injection volume (partial loop mode) on to a Hypersil Gold C18, (100 mm × 2.1 mm, 1.9 μm particle size) UHPLC column (Thermo-Fisher Ltd.) with the Dionex U3000 UHPLC system coupled to a Thermo LTQ-FT-MS Ultra system (Thermo-Fisher Ltd.). Solvent A, HPLC grade water, and solvent B, HPLC grade methanol (J.T. Baker, UK) were acidified with 0.1% formic acid (Aristar grade, VWR Ltd., UK). The gradient program was as follows: hold 100% A 0–1 min, 100% A – 100% B 1–3.5 min curve 3, hold 100% B 3.5–6 min, 100% B – 100% A 6–7 min curve 3, hold 100% A 7–8 min. The LTQ-FT-MS Ultra system was operated under Xcalibur software (Thermo-Fisher Ltd.), in full scan mode (*m/z* 100–1000) at a mass resolution of 50,000 (FWHM defined at *m/z* 400). Prior to the analytical run, the LTQ and FT-MS were calibrated with the manufacturers recommended calibration mixture. The samples were analyzed in a completely randomized order. A blank control sample was analyzed at the start and end of the run, thus providing a measure of the sample background and also a measure of compound carry over. The relative peak areas of 3-hydroxy nonanoic acid (monoisotopic mass: 174.125595 Da, retention time (RT) 3.7 min) were obtained for each sample and the 3-hydroxy nonanoic acid analytical standard in the Qual Browser function of the Xcalibur software package (Thermo-Fisher Ltd.). The peak areas were calculated based upon the EIC for the major ESI negative mode base peak, 173.1182 *m/z* (the deprotonated parent ion M-H), which was detected at a RT of 3.7 min. Peak areas were exported to Microsoft Excel, the standard deviation was calculated across the replicates within each of the defined experimental classes. Finally graphs were generated where error bar was representative of the standard deviation upon peak area.

Lipids were like-wise extracted from *C. neoformans* LMPE 046 [cell density = 1.9 × 10^7^ cells/ml (±3.0 × 10^5^)] and *Acanthamoeba castellanii* LMPE 187 [cell density = 1.8 × 10^5^ cells/ml (±2.0 × 10^3^)] and analyzed on a ABSCIEX 3200 QTRAP hybrid triple quadrupole ion trap mass spectrometer (Toronto, ON, Canada) with an Agilent 1200 SL HPLC stack as a front end. All data acquisition and processing was performed using Analyst 1.5 (ABSCIEX) software. Twenty microliter of each extracted sample was separated on a C18 (50 mm × 4.6 mm, XDB-C18, Agilent) column at a flow rate of 300 μl/min using an isocratic 90:10 [MeOH/0.1% formic acid: H_2_O/0.1% formic acid (Merck, South Africa)] solvent composition for a total 3 min analysis time in positive mode. During initial method optimization it was found that the analyte precursor ionizes in both positive and negative mode on this instrument but yielded better MRM transitions in positive only mode. Eluting analytes were ionized by electrospray in the TurboV ion source with a 400°C heater temperature to evaporate excess solvent, 20 psi nebuliser gas, 20 psi heater gas and 20 psi curtain gas and the ion spray voltage was set at 5500 V. To analyze the samples, a targeted Multiple Reaction Monitoring (MRM) workflow was performed. The targeted analyses of 3-hydroxy nonanoic acid were performed using five MRM transitions [175.1 > 139.3 (quantifier); 175.1 > 97.2; 175.1 > 55.1; 175.1 > 69.1; 175.1 > 121.1 (qualifiers)]. The peak area on the chromatogram generated from the first and most sensitive transition was used as the quantifier while the other transitions are used as qualifiers. The qualifiers serve as an additional level of confirmation for the presence of the analyte, the RT for these two transitions needs to be the same. The EIC from the quantifier transition is shown as supplementary data for comparison.

### Visualization of *Cryptococcus*-amoeba Interactions: Fluorescent Microscopy and Transmission Electron Microscopy

Fluorescent images were taken after cryptococcal cells (*C. neoformans* UOFS Y-1378) were stained with fluorescein isothiocyanate (Sigma–Aldrich, South Africa; 1 μl of stain : 999 μl of cells) for 2 h at room temperature, while at the same time amoeba cells (100 μl) were allowed to adhere to wells on a chamber slide (Nunc^®^ Lab-Tek^®^ II Chamber Slide^TM^ system; Sigma–Aldrich) at 30°C. After this 2 h-period, cryptococcal cells were washed twice with PBS and added 100 μl to the chamber slide (for 2 h at 30 C) in order to interact with amoeba cells (10 amoebae: 1 fungus). At the end of the interactive period, wells were washed twice with PBS to remove any cryptococcal cells not internalized. The slide was then fixed for 1 h with 2.5% glutaraldehyde (Sigma–Aldrich) following which, the fixative was aspirated. An antifade compound, 1,4-diazabicyclo[2.2.2]-octane (Sigma–Aldrich), was added to the slide before viewing using a confocal laser scanning microscope (CLSM; Nikon TE 2000; Tokyo, Japan). Material for transmission electron microscopy (TEM) was obtained from a 48 h co-culture [1 ml: 1 ml (v/v) of 10 amoebae : 1 fungus (*C. neoformans* UOFS Y-1378)] that was grown at 30°C. The material was prepared for TEM viewing according to the method of van Wyk and Wingfield (1991). In brief, this co-cultured material was chemically fixed with 1.0 M (pH 7) sodium phosphate-buffered gluteraldehyde (3%) for 3 h and then for 1.5 h in similarly buffered osmium tetroxide. The material was next dehydrated in a graded acetone series. The TEM material was then embedded in epoxy resin and polymerized at 70°C for 8 h. An LKB III Ultratome was used to cut 60-nm sections with glass knives. Uranyl acetate was used to stain sections for 10 min, followed by lead citrate for 10 min and the preparation viewed with a Philips EM 100 transmission electron microscope (van Wyk and Wingfield, 1991).

### *Cryptococcus* Phagocytosis Assay

We assessed the ability of amoebae to internalize cryptococcal cells: (1) obtained from strains *C. neoformans* UOFS Y-1378, (2) *C. gattii* R265, (3) *C. neoformans* LMPE 046 in the absence or presence of 3-hydroxy C9:0, i.e., 0 mM, 0.2 and 1 mM, using the phagocytosis stain, pHrodo^TM^ Green Zymosan A BioParticles (Life Technologies, US). The stain only fluoresces when excited at acidic pH, such as inside a food vacuole or phagosome. Cryptococcal cells were standardized to 1 × 10^6^ cells/ml in PBS (which has a neutral pH) and stained (1 μl of stain: 999 μl of cells) for 1 h at room temperature while slowly agitating. Next, cryptococcal cells were washed with PBS, spun down and suspended in sterile 1000 μl of PBS. A 100-μl suspension of cells was then transferred to microtitre plate wells (Greiner Bio-One, Germany) and allowed to interact with amoebae (100 μl; 1 × 10^5^ cells/ml) for 2 or 6 h at 30°C. The amoebae were standardized in fresh PYG broth (pH 7). At the end of the incubation period, the induced fluorescence was measured (492 nm; ex/538 nm; em) using a Fluoroskan Ascent FL (Thermo-Scientific, USA) microplate reader, which converts logarithmic signals to relative fluorescence units. The fluorescence was also measured for fungal cells alone in order to normalize the readings.

### *Cryptococcus* Survival Assay

The interactive outcome of amoeba cells and fungal cells was quantified by enumerating viable fungal cells by counting colony forming units (CFU). The above was based on a modified protocol previously detailed by Steenbergen et al. (2001). Here, cryptococcal cells, i.e., obtained from strains *C. neoformans* UOFS Y-1378, *C. gattii* R265 and *C. neoformans* LMPE 046, were added to amoeba cells in the absence or presence of 3-hydroxy C9:0 (0 and 0.2 mM) to yield a ratio of 10 (amoebae): 1 (fungus), i.e., 500 μl: 500 μl (v/v). These cultures were grown at 30°C in 1.5 ml eppendorf tubes (Merck). After a 48 h interactive period, co-cultured cells were gently agitated and amoeba cells were lysed by forcibly pulling and pushing them through a needle (27 gage × 20 mm; Novagen, South Africa) eight times (Steenbergen et al., 2001). For each tube, serial dilutions were made and plated out on YPD agar plates for 48 h at 30°C. Additionally in a separate experiment, we determined the susceptibility levels of *C. neoformans* UOFS Y-1378 cells toward amoebae in the absence of 3-hydroxy fatty acids. To be specific, *C. neoformans* UOFS Y-1378 cells were initially treated with 1 mM aspirin, which is an inhibitor of 3-hydroxy fatty acids (Sebolai et al., 2008). Following a 48 h aspirin-treatment period, cells were then fed to amoebae at a ratio of 10 (amoebae): 1 (fungus). This co-culture was incubated as stated above and cryptococcal cells were enumerated in the same manner.

### The Effect of cryptococcal 3-hydroxy Fatty Acids on Amoebae

A 100-μl suspension of amoeba cells concentrated to 10^5^ cells/ml in PYG broth was added to wells of a sterile, disposable 96-well flat-bottom microtitre plates. Thereafter, aliquots of 100 μl of the test drug (3-hydroxy C9:0), at twice the desired final concentrations, were dispensed into wells. To the point, cells were tested at final concentrations of 0.2 and 1 mM of 3-hydroxy fatty acids. Amoeba cells were also tested against nonanoic acid (C9:0) at the same concentrations. The plates were then incubated for 48 h at 30°C. At the end of the incubation period, cells were reacted in the dark at 30°C with 2,3-bis-(2-methoxy-4-nitro-5-sulfophenyl)-2*H*-tetrazolium-5-carboxanilide (XTT; Sigma–Aldrich) in the presence of menadione (Sigma–Aldrich) – in order to measure their metabolic activity (Polat et al., 2014). The optical density (OD) readings were measured, after 3 h of initiating the tetrazolium reaction, using a spectrophotometer (Biochrom EZ Read 800 Research, UK). Non-treated amoeba cells were included for reference.

### Statistical Note

All experiments, reported in this study, were performed in triplicate. And where appropriate, a student *t*-test was conducted to determine the statistical significance of data between the different experimental conditions.

## Results

### Characterization of Cryptococcal 3-hydroxy Fatty Acids

**FIGURE 1:**
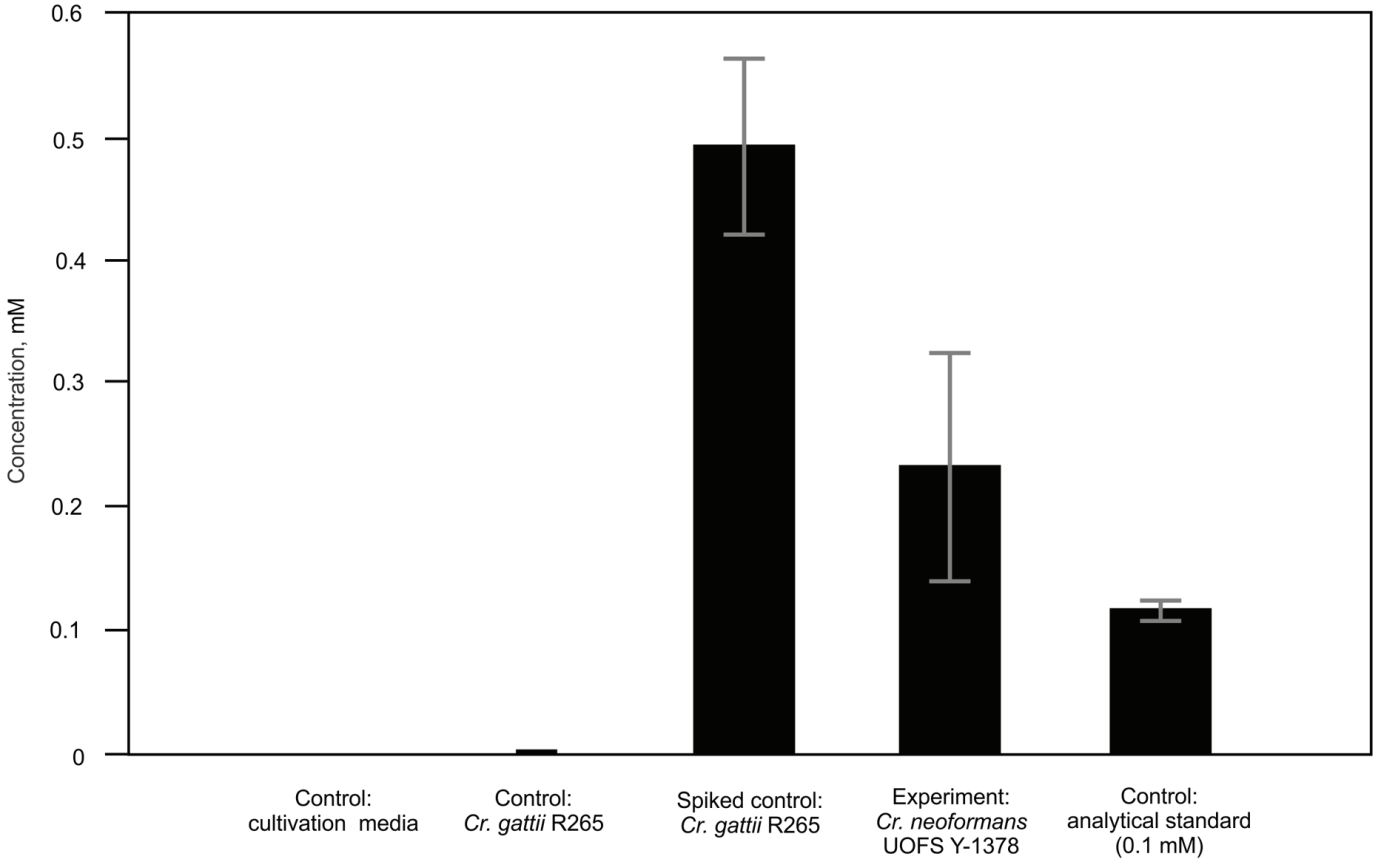
**Estimation of the physiological concentrations of 3-hydroxy nonanoic acid produced by *Cryptococcus neoformans* UOFS Y-1378.** Using a two-point calibration method, we extrapolated the secreted concentration to be in the range: 0.1–0.4 mM. For each sample class, five biological replicates were analyzed and the values are the mean with standard deviation indicated by error bars. *C. gattii* was spiked with the analytical standard solution at a final concentration of 0.66 mM.

We previously assigned a structure of a 3-hydroxy fatty acid-based molecule (after detection with a 3*R*-hydroxy fatty acid-specific polyclonal antibody and initial GC-MS analysis) to a hydroxy fatty acid extracted from *C. neoformans* UOFS Y-1378 cultures (Sebolai et al., 2007). However, in order to determine the biological function(s) of these capsule-associated molecules, it is important to know their secreted concentration. Therefore, in order to estimate concentrations produced by *C. neoformans* UOFS Y-1378, the extracted ion chromatograms (EICs) were compared between (1) the biological samples of *C. neoformans* UOFS Y-1378, (2) biological samples of *C. gattii* R265 that were spiked with 3-hydroxy nonanoic acid to a final concentration of 0.66 mM, and (3) a 0.1 mM solution of the 3-hydroxy nonanoic acid dissolved in water. Using a two-point calibration method, our analysis indicated that the biological sample concentration range of 3-hydroxy nonanoic acid was in the range 0.1–0.4 mM (**Figure 1**). Although these estimates were based upon comparisons to a single concentration level of the analytical standard rather than a full dilution series based calibration curve and matrix-matched standards – the extrapolated figures are sufficient for providing a concentration range that is suitable for conducting biological studies.

**FIGURE 2:**
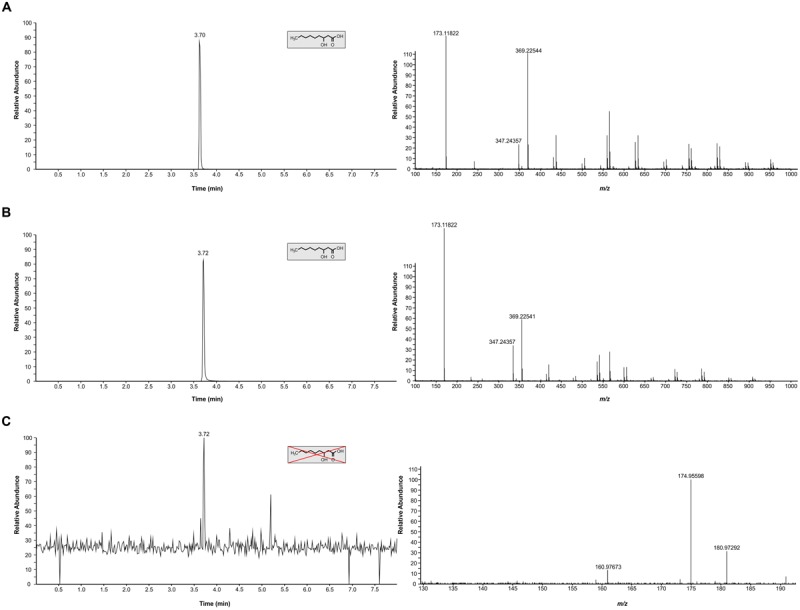
**Characterization of 3-hydroxy fatty acids in *C. neoformans* UOFS Y-1378 and *C. gattii* R265. (A)** The EIC (top left graphic) obtained for the analytical standard compound (3-hydroxy nonanoic acid) and its corresponding mass spectrum (top right graphic). **(B)** The EIC (middle left graphic) obtained for *C. neoformans* analyte and its corresponding mass spectrum (middle right graphic). *C. neoformans* had a similar profile (i.r.o. retention time and diagnostic peaks) suggesting this fungus produces 3-hydroxy nonanoic acid. **(C)** The EIC (bottom left graphic) obtained for *C. gattii* and its corresponding mass spectrum (bottom right graphic). *C. gattii* produced an unknown metabolite, which had a similar retention time to the analytical standard compound. However, we could not detect diagnostic peaks ([M-H]^-^, [M + Na + M-H]^-^ and [M + M-H]^-^) that are characteristic of our metabolite of interest (3-hydroxy nonanoic acid).

In light of our comparative studies, it was also important to establish if *C. gattii* R265 produced the same metabolite or not. Here, we analyzed the authentic chemical standard for 3-hydroxy nonanoic acid and biological samples and matched the RT (3.7 min and accurate *m/z* (173.1182; [M-H]^-^) between standard and samples (**Figures 2A–C**). For *C. neoformans* UOFS Y-1378, as expected, we observed a similar MS/MS mass spectrum for the standard and samples showing the detection of a hydrogen-bound dimer ion ([M + M-H]^-^), and a sodium-bridged dimer ion ([M + Na + M-H]^-^) and in the same response ratios (**Table 1**; **Figures 2A,B**). With respect to *C. gattii* R265, we also noted elution of an unknown metabolite at a RT that matched the analytical standard, although at very low levels approaching the limit of Fourier transform-ion cyclotron resonance-mass spectrometer detection. However, upon studying its mass spectrum, this metabolite did not have the characteristic diagnostic MS/MS peaks of the chemical standard or the metabolite of interest (**Table 1**; **Figures 2A,C**). *C. neoformans* LMPE 046 and *Acanthamoeba castellanii* LMPE 187 were also shown to not produce any 3-hydroxy fatty acids (**Supplementary Figure S1**). Both their respective EICs did not show elution of our metabolite of interest after 2.05 min when referenced against the EIC of the analytical standard compound.

**Table 1:** Molecule annotation of the cryptococcal 3-hydroxy fatty acid molecule based on the comparison of retention time (RT) and accurate *m/z* determinants.

Sample details		Characterization of 3-hydroxy fatty acid extracts				
Experimental class	Replicates	RT (min)	^∗^Diagnostic MS/MS peaks			^†^Analyte structure
			[M-H]^-^	[M + M-H]^-^	[M + Na + M -H]^-^	
Analytical standard	*n* = 5	3.70	173.118	347.244	369.225	3-Hydroxy C9:0
*Cryptococcus neoformans*	*n* = 5	3.72	173.118	347.244	369.225	3-Hydroxy C9:0
*Cryptococcus gattii*	*n* = 5	3.72	–	–	–	Unknown

*[M-H]^-^ = Deprotonated molecular ion; [M + M-H]^-^ = Hydrogen-bound dimer ion; and [M + Na + M-H]^-^ = Sodium-bridged dimer ion. ^†^3-Hydroxy C9:0 = 3-Hydroxy nonanoic acid.

### Visualization of *Cryptococcus*–amoeba Interaction

**FIGURE 3:**
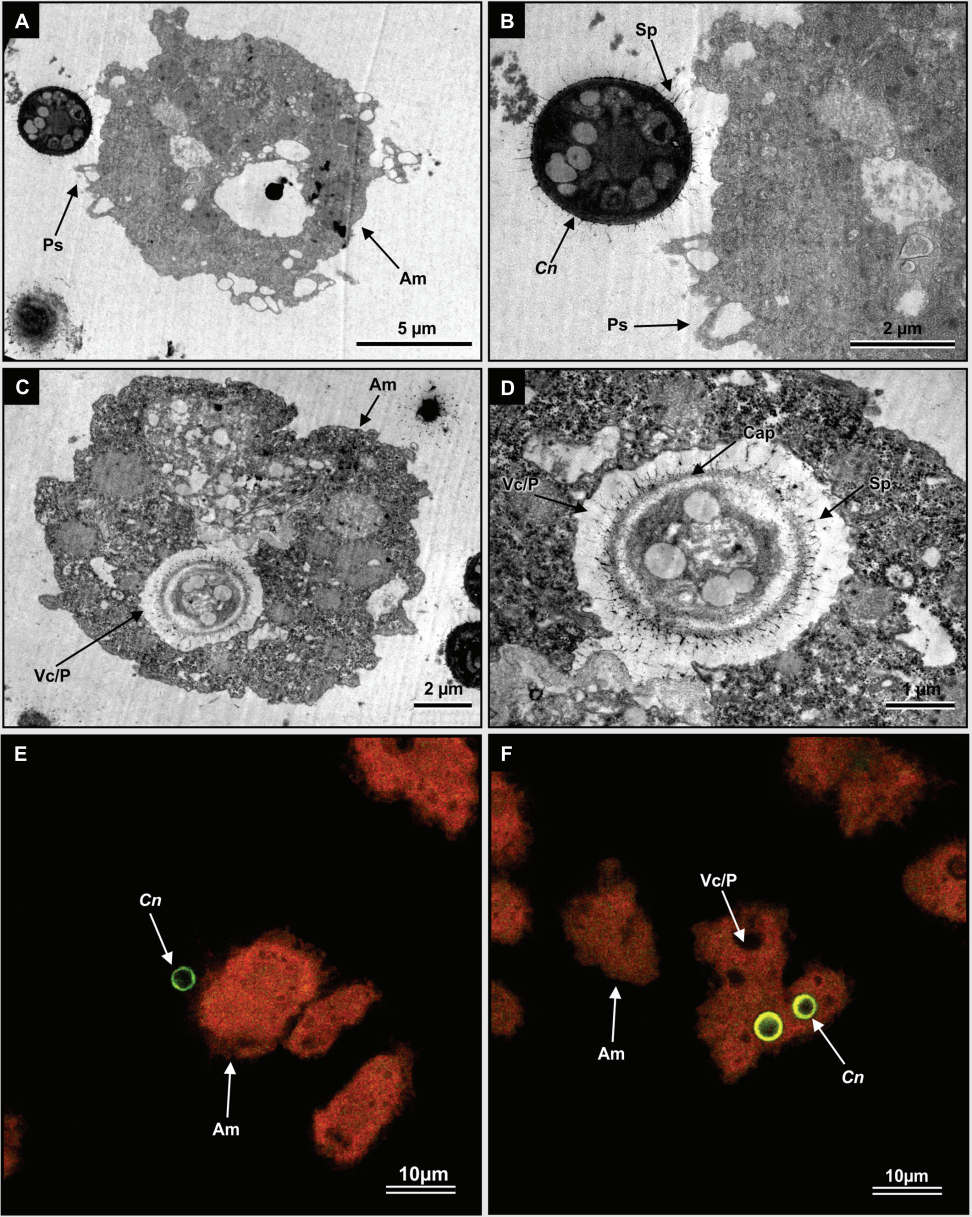
**The visualization of interactive moments between *C. neoformans* UOFS Y-1378 cells and amoebae, *Acanthamoeba castellanii* LMPE 187. (A)** Shows a cryptococcal cell in close proximity to an amoeba cell. **(B)** Is an enlargement of **(A)** showing pseudopodia about to catch a cryptococcal cell. **(C)** Shows a cryptococcal cell, with characteristic capsular protuberances, that has been internalized while **(D)** is an enlargement of an internalized cell inside the food vacuole. **(E)** And **(F)** are fluorescent micrographs, which show internalized *Cryptococcus* cells (depicted in a green-color) inside an autofluorescing amoebae (depicted in an orange color). Am, amoeba; C, *Cryptococcus*; Cap, capsule; Fv/p, food vacuole or phagosome; Ps, pseudopodia; Sp, spiky protuberances.

Cryptococcal cells often fall prey to foraging amoebae in nature. In order to reproduce a similar setting *in vitro, C. neoformans* UOFS Y-1378 was fed to *Acanthamoeba castellanii* LMPE 187, in order to view their interactions. Transmission electron micrographs revealed a moment when a cryptococcal cell was about to be captured by amoeba pseudopodia (**Figures 3A,B**) and after being trapped inside a food vacuole or phagosome (**Figure 3C**). The characteristic thick capsule, with the typical spiky protuberances of *C. neoformans* UOFS Y-1378, can clearly be seen in **Figure 3D**. We previously suggested these spiky protuberances may facilitate the release of 3-hydroxy fatty acids into the extracellular environment after detecting their presence inside protuberances following TEM immuno-gold labeling analysis (Sebolai et al., 2008). The fluorescent micrographs provide further pictorial evidence of a cryptococcal cell (in green) close to amoeba (in orange) (**Figure 3E**) and internalized cryptococcal cells (in green) (**Figure 3F**). It is reasonable to conclude that during such interactive moments, the source of 3-hydroxy fatty acids can only be *C. neoformans* UOFS Y-1378 (**Figure 2B**) and not amoebae (**Supplementary Figure S1**) as per the LCMS results.

### 3-Hydroxy Fatty Acids Protect Cells from Amoebal Phagocytosis

**FIGURE 4:**
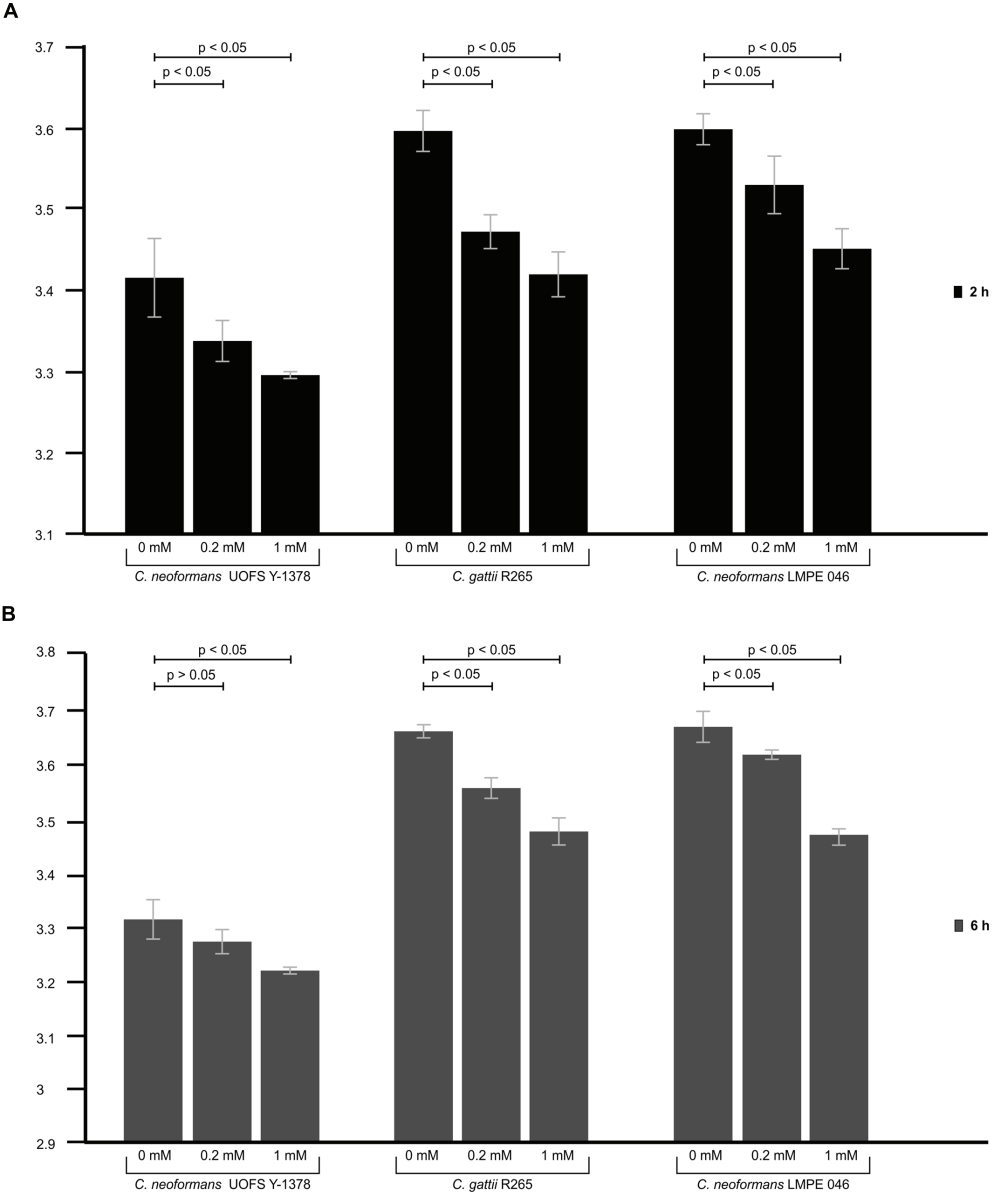
**The results of the phagocytosis assay of cryptococcal cells co-cultured with amoeba.** Through using the phagocytosis stain, pHrodo^TM^ Green Zymosan A BioParticles, the appetite of amoebae to internalize *C. neoformans* UOFS Y-1378, *C. neoformans* LMPE 046 and *C. gattii* R265, in the presence and absence of 3-hydroxy fatty acids, was measured after 2 h **(A)** and 6 h **(B)** of co-culturing. At both time intervals, *C. neoformans* UOFS Y-1378 cells were more resistant (*p* < 0.05) to amoebae compared to *C. neoformans* LMPE 046 cells and *C. gattii* R265 cells based on the recorded relative fluorescence units. Addition of 3-hydroxy fatty acids to *C. neoformans* UOFS Y-1378, *C. neoformans* LMPE 046 and *C. gattii* R265 cells, at both 2 and 6 h, seem to have significantly (*p* < 0.05) decreased their vulnerability toward amoebae when compared to *C*. *neoformans* UOFS Y-1378, *C. neoformans* LMPE 046 and *C. gattii* R265 cells in the absence of 3-hydroxy fatty acids.

In the absence of artificially added 3-hydroxy fatty acids, the test amoeba strain yielded significantly lower relative fluorescence units (*p* < 0.05) when co-cultured with *C. neoformans* strain UOFS Y-1378 compared to when co-cultured with *C. gattii* R265 and *C. neoformans* LMPE 046 at both 2 h (**Figure 4A**) and 6 h (**Figure 4B**). The latter implies that amoeba displayed less appetite to internalize *C. neoformans* UOFS Y-1378, which naturally produces 3-hydroxy fatty acids, when compared to *C. gattii* R256 and *C. neoformans* 046, which both do not. In order to investigate if 3-hydroxy fatty acids may be responsible for the displayed resistance expressed by *C. neoformans* UOFS Y-1378, we re-assessed the appetite of amoebae for *C. gattii* R256 cells and *C. neoformans* LMPE 046 cells when 3-hydroxy fatty acids were artificially added, i.e., 0.2 and 1 mM, to their culture media. Here, addition of 3-hydroxy fatty acids made *C. gattii* R256 and *C. neoformans* LMPE 046 more resistant to amoebal internalization or less appetizing to be internalized at both 2 and 6 h in a dose-dependent manner – as per the recorded lower relative fluorescence units when compared to higher readings obtained for *C. gattii* R256 and *C. neoformans* LMPE 046 in the absence of 3-hydroxy fatty acids (**Figure 4**). Additionally, *C. neoformans* UOFS Y-1378 also displayed a dose-dependent resistance toward being internalized by amoebae when increasing amounts of 3-hydroxy fatty acids were artificially added. It is worthwhile to note that the number of *C. neoformans* UOFS Y-1378 cells that were internalized (in the absence and presence of 3-hydroxy fatty acids) generally decreased over time. While on the other hand, the number of *C. gattii* R256 cells and *C. neoformans* 046 cells that were internalized (in the absence and presence of 3-hydroxy fatty acids) generally increased over time. It is also interesting to note that for each co-culture experiment, and specifically at 6 h, the number of internalized cryptococcal cells in the presence of 3-hydroxy fatty acids, were approximate to the number of internalized cryptococcal cells in the absence of 3-hydroxy fatty acids compared to at 2 h. This gap-narrowing or approximation suggest a level of adaptation by amoebae, over time, to the presence of 3-hydroxy fatty acids.

**FIGURE 5:**
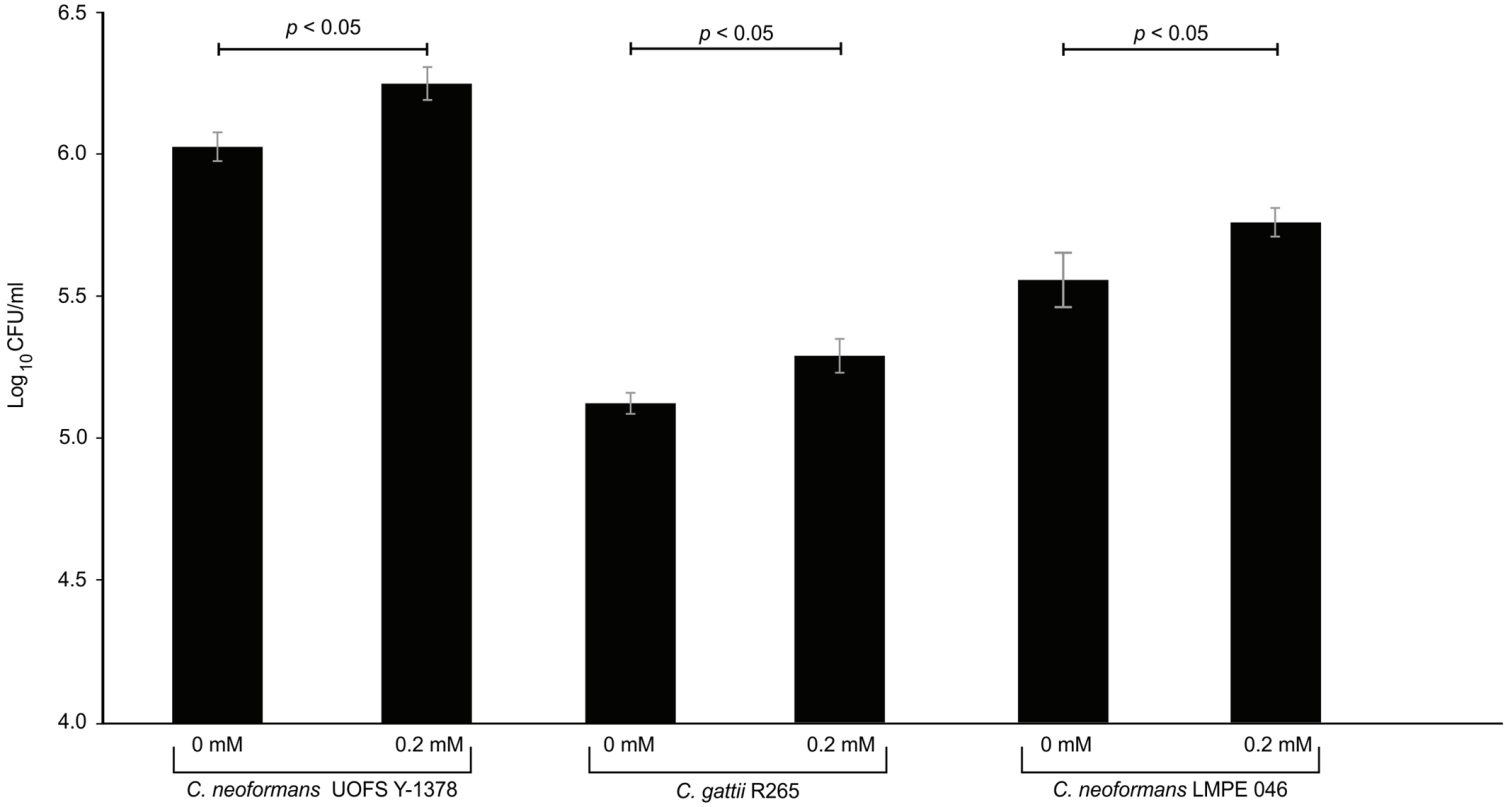
**The results of the survival assay of cryptococcal cells co-cultured with amoeba.** The results obtained corroborate the phagocytosis assay outcome. As expected, *C. neoformans* UOFS Y-1378 was naturally resistant to amoebae and thus more cells (*p* < 0.05) were recovered on agar plates compared to recovered cells of *C. neoformans* LMPE 046 and *C. gattii* R265. The addition of 3-hydroxy fatty acids to cultures of *C. neoformans* UOFS Y-1378, *C. neoformans* LMPE 046 and *C. gattii* R265 made these cells also resistant to amoebae.

**FIGURE 6:**
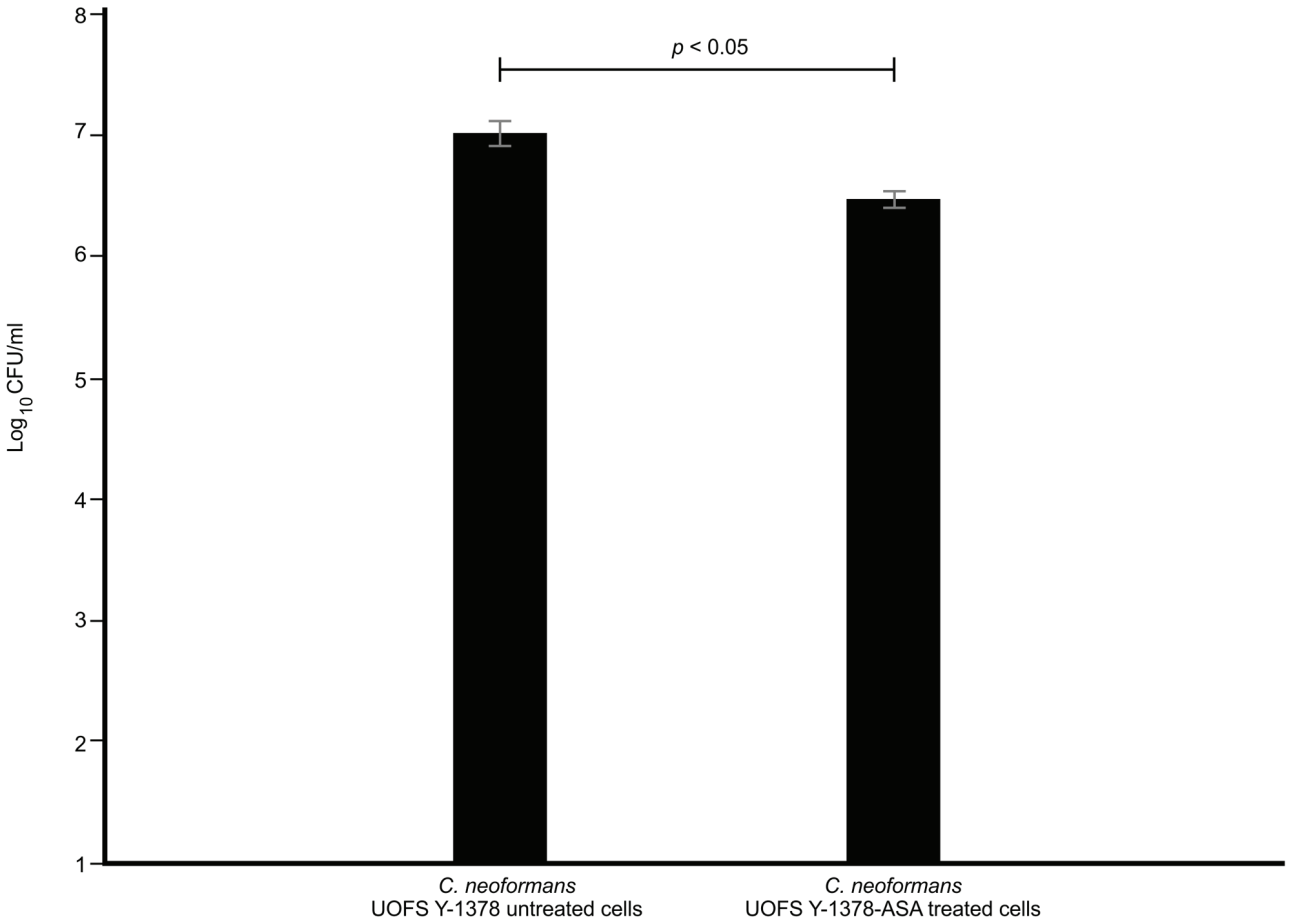
**The results of the survival of aspirin-treated *C. neoformans* UOFS Y-1378 cells and non-treated *C. neoformans* UOFS Y-1378 cells co-cultured with amoeba.** The inhibition of 3-hydroxy fatty acids by aspirin made *C. neoformans* more susceptible (*p* < 0.05) to amoebae as less cells were recovered on agar plates when compared to non-treated cells.

Next, we quantified the survival of fungal cells after being internalized. And as expected (in the absence and presence of 3-hydroxy fatty acids), *C. neoformans* UOFS Y-1378 was more resistant to amoebae, i.e., few cells were successfully phagocytosed, compared to *C. gattii* R256 and *C. neoformans* LMPE 046 as per the number of recovered fungal colonies on agar plates (**Figure 5**). With respect to *C. neoformans* UOFS Y-1378, the observed phagocytosis outcome suggests that of the few cells that were successfully internalized (according to **Figure 4** results); more of them were able to survive the phagocytic process and the opposite phenomenon is observed with respect to *C. gattii* R256 cells and *C. neoformans* LMPE 046 cells. This determination suggests that in addition to impairing cell internalization, this molecule may also promotes intracellular survival. Once more, the addition of 3-hydroxy fatty acids to *C*. *neoformans* UOFS Y-1378, *C. gattii* R256 and *C. neoformans* LMPE 046 culture media significantly (*p* < 0.05) increased their level of resistance toward amoebae by promoting intracellular survival. It is also striking to note that inhibition of 3-hydroxy fatty acid production using aspirin resulted in *C. neoformans* UOFS Y-1378 cells being more susceptible to amoebae compared to non-treated *C. neoformans* UOFS Y-1378 cells (**Figure 6**). In 2008, we showed that 3-hydroxy fatty acids are inhibited in a dose-dependent manner by increasing amounts of aspirin. Taken together, the above data points toward a possible protective quality that is exhibited by 3-hydroxy fatty acids.

### 3-Hydroxy Fatty Acids Effect on Amoebae

**FIGURE 7:**
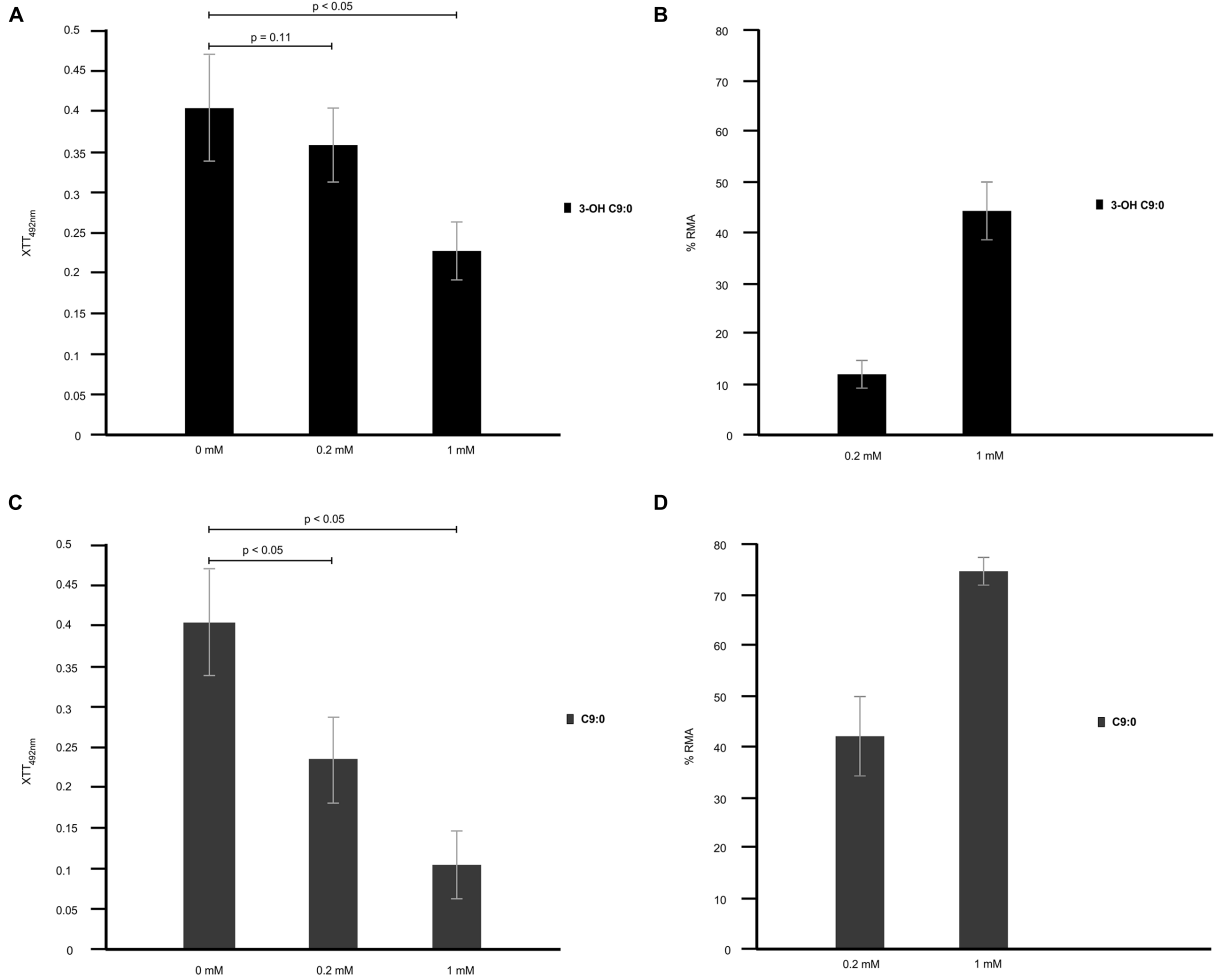
**The cytotoxicity effect of 3-hydroxy fatty acids on amoebae. 3-Hydroxy nonanoic acid (3-OH C9:0) was shown to be less effective as an anti-amoebae agent **(A,B)** when compared to nonanoic acid (C9:0) **(C)**, which yielded a higher percentage reduction in metabolic activity (% RMA) **(D)**.** At the estimated physiological concentration of 0.2 mM, 3-hydroxy nonanoic acid yielded XTT reduction readings that were highly comparable (*p* = 0.11) to readings obtained for non-treated amoebae after a 48 h period. The latter suggest that this molecules does not negative affect the growth of amoebae.

Exposure of amoeba cells to estimated physiological concentration of cryptococcal 3-hydroxy fatty acids, i.e., 0.2 mM, yielded XTT reduction values that were highly comparable (*p* = 0.11; not-significant) to reading obtained for non-treated amoeba cells. However, at 1 mM concentration, the XTT reduction values were significantly (*p* < 0.05) lower compared to that of non-treated amoeba cells (**Figure 7A**). Next, these XTT reduction values were translated into percentage reduction in metabolic activity (% RMA) in order to determine the measure of cytotoxicity exerted by 3-hydroxy fatty acids on amoebae. Toward this end, 0.2 mM yielded 12% RMA while 1 mM yielded 43% RMA (**Figure 7B**). These findings suggest that 3-hydroxy fatty acids do not negatively affect the growth of amoebae, more so at estimated physiological concentrations; and this crucial as cryptococcal cells require a viable host for intracellular survival. Moreover, the former also suggests that amoebae do not metabolize 3-hydroxy fatty acids to support their growth, as addition of 3-hydroxy C9:0 to the culture media did not increase their metabolic activity. This further supports the argument that 3-hydroxy fatty acids may be particularly secreted to impair the phagocytic machinery of amoeba. When considering the effect of nonanoic acid (C9:0; which is structurally close to 3-hydroxy C9:0) on amoeba’s metabolic activity, this molecule was observed to exert a greater negative effect on the metabolic activity of amoeba cells at 0.2 mM (*p* < 0.05) and 1 mM (*p* < 0.05) compared to 3-hydroxy fatty acids (**Figure 7C**). The latter also translated into significantly higher %RMA at 0.2 mM (42%) and 1 mM (73%) compared to 3-hydroxy C9:0 (**Figure 7D**).

## Discussion

*Cryptococcus* species are reported to act as parasites of phagocytic cells wherein they can successfully establish an intracellular lifestyle (Levitz et al., 1999; Heitman et al., 2011) and in some instances, use phagocytic cells to disseminate to other organs (Voelz and May, 2010). To date, the capsule has been credited with shielding cells against the effects of phagocytic cells. This study shows that in addition to capsules, 3-hydroxy fatty acids also have a role to play possibly in concert with the capsule. To the point, our findings suggest that 3-hydroxy fatty acids are secreted at a high enough concentration to display a protective quality that prevents internalization and possibly promote intracellular survival. Importantly, at this concentration, the molecule does not kill or negatively affect its host cell and this is paramount to any parasite’s quest to successfully establishing an intracellular lifestyle.

Phagocytosis is a receptor-mediated process that is governed by a balance between pro- and anti-signal molecules that promote or inhibit the process of phagocytosis (Freeman and Grinstein, 2014). To be internalized, a cell must first bind to the surface of amoeba. However, as Bottone et al. pointed out, not all cells that are bound are internalized and eventually phagocytosed by amoebae (Bottone et al., 1994). The latter may be driven, for example, by antagonistic microbial secretomes that inhibit microbial recognition. Toward this end, 3-hydroxy fatty acids could impair the intracellular signaling mechanism that is required to initiate phagocytosis. To ascertain this, further studies are, however, required in order to understand the molecular mechanism(s) that confer the protective quality of cryptococcal 3-hydroxy fatty acids. The idea of lipids being trafficked into the extracellular environment of microbes including *C. neoformans*, has previously been reported on by Rodrigues et al. (2007). In their paper, these authors point out that upon release – the secreted molecules can mediate host–pathogen interactions in favor of the pathogen leading to a diseased-state. Importantly, they highlight lipids such as phosphatidylserine that may decrease microbial killing – thus promoting pathogenesis (Rodrigues et al., 2007). It is also not surprising that our 3-hydroxy fatty acid may impair the phagocytic machinery of amoebae as the role of fatty acids in preventing phagocytosis is well documented. In one study, short chained fatty acids were reported to prevent the release of lysozymes (Eftimiadi et al., 1987), and in other another, fatty acids reduced hydrogen peroxide roduction thus impairing phagocytosis (Bellinati-Pires et al., 1993).

It was encouraging to also observe that inhibition of cryptococcal 3-hydroxy fatty acids made cells more susceptible to amoebal phagocytosis. Aspirin inhibits of 3-hydroxy fatty acid production by outcompeting the product of 3-hydroxyacyl-CoA dehydrogenase activity in the mitochondria as a result of structural similarities between aspirin’s active metabolite, salicylate, and the product (Glasgow et al., 1999). It will be interesting to determine if treatment of cryptococcal cells with aspirin will also chemosensitise macrophages to successfully phagocytose internalized cells as we have shown. However, when using aspirin, caution should be taken to realize the desired outcome to the exclusion of adverse effects. In this study, we tested aspirin at a concentration, 1 mM, which is considered as optimum for safe and effective therapy in the blood (Levy, 1976). The latter thus points to the possible application of aspirin as a candidate drug that can prevent cryptococcal cells from establishing an intracellular lifestyle.

## Author Contributions

All authors contributed significantly to the paper and all authors are in agreement with the content of the manuscript. OS is the principal investigator and designed and coordinated the study. UM and OS performed the experiments. AO, BM, CP, JA, and CS gave strategic inputs. WD and RM provided funding and availed their facilities for the UK-based studies. JWA and AS assisted with LCMS analysis. UM, AO, BM, CP, JA, CS, JWA, AS, WD, RM, and OS wrote the manuscript.

## Conflict of Interest Statement

The authors declare that the research was conducted in the absence of any commercial or financial relationships that could be construed as a potential conflict of interest.

## Supplementary Material

Figure S1 **Determination of 3-hydroxy fatty acids production by cultures of *C. neoformans* LMPE 046 and *Acanthamoeba castellanii* LMPE 187. (A)** The EIC obtained for the analytical standard compound (3-hydroxy nonanoic acid) showing elution of our metabolite of interest after 2.05 min. **(B)** The EICs obtained for *C. neoformans* LMPE 046 and **(C)** *Acanthamoeba castellanii* LMPE 187 showing no elution of 3-hydroxy nonanoic acid after 2.05 min, suggesting these species do not produce 3-hydroxy nonanoic acid.
