# Supplementary figures and images for: Cryptococcal 3-Hydroxy Fatty Acids Protect Cells Against Amoebal Phagocytosis
Source: Front Microbiol. 2015 Dec 9;6:1351. doi: 10.3389/fmicb.2015.01351 (PMC4673343; doi:10.3389/fmicb.2015.01351)

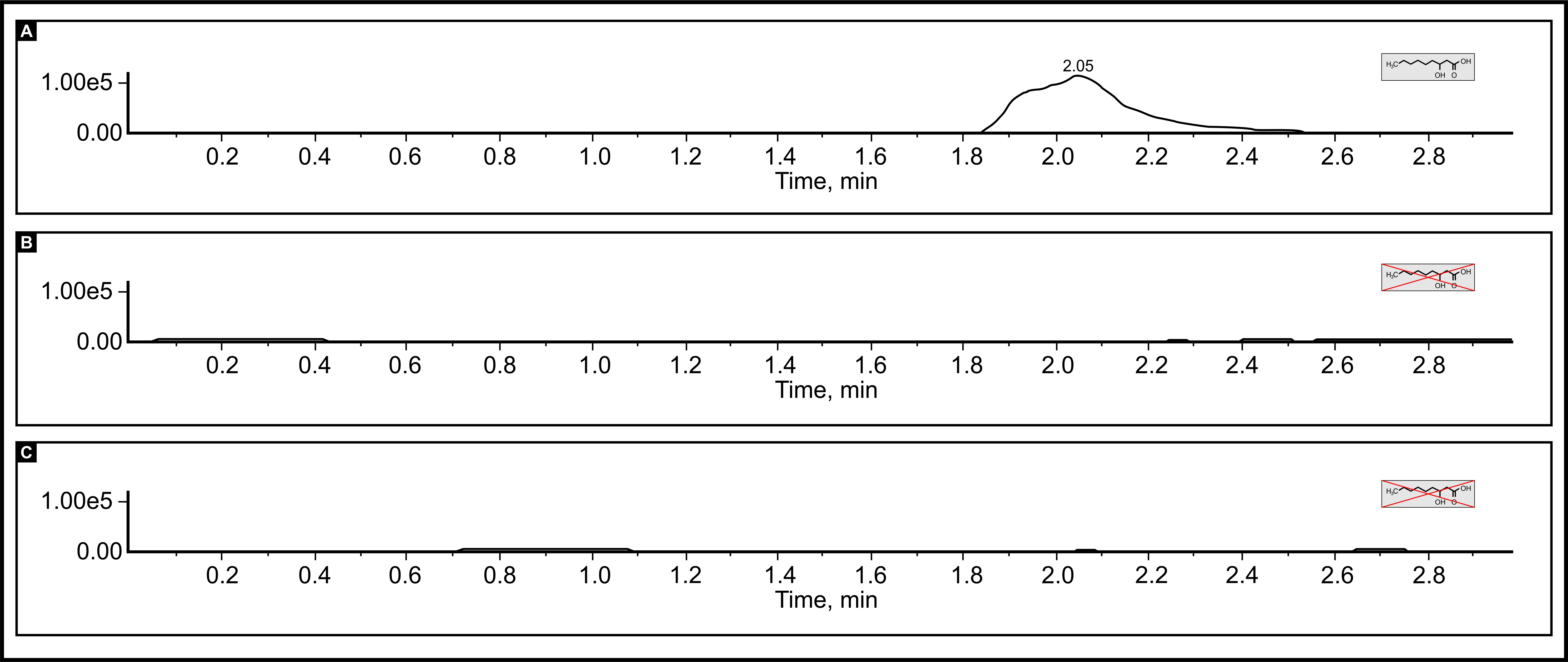

Supplement: Figure S1 — Determination of 3-hydroxy fatty acids production by cultures of C. neoformans LMPE 046 and Acanthamoeba castellanii LMPE 187. (A) The EIC obtained for the analytical standard compound (3-hydroxy nonanoic acid) showing elution of our metabolite of interest after 2.05 min. (B) The EICs obtained for C. neoformans LMPE 046 and (C) Acanthamoeba castellanii LMPE 187 showing no elution of 3-hydroxy nonanoic acid after 2.05 min, suggesting these species do not produce 3-hydroxy nonanoic acid. [file Image_1.JPEG]
